# Atypical presentations of UTUC: a case report of three patients

**DOI:** 10.3389/fonc.2023.1294316

**Published:** 2024-01-08

**Authors:** Linfa Guo, Xiaojie Bai, Kuerban Tuoheti, Xiaolong Wang, Tongzu Liu

**Affiliations:** ^1^ Department of Urology, Zhongnan Hospital of Wuhan University, Wuhan, China; ^2^ Cancer Precision Diagnosis and Treatment and Translational Medicine Hubei Engineering Research Center, Zhongnan Hospital of Wuhan University, Wuhan, China; ^3^ Hubei Province Key Laboratory of Urinary System Diseases, Zhongnan Hospital of Wuhan University, Wuhan, China

**Keywords:** upper tract urothelial carcinoma, misdiagnosis, white blood cell count, fever, case report

## Abstract

**Background:**

Upper tract urothelial carcinoma (UTUC) is a rare clinical condition primarily characterized by symptoms such as gross or microscopic hematuria, flank pain, and renal colic. Although computed tomography urography (CTU) is currently the most accurate imaging modality for diagnosis, atypical presentations and physical examination findings can sometimes obscure lesions, posing diagnostic challenges.

**Case presentation:**

In this report, three patients exhibited atypical symptoms, sharing a common complaint of flank pain. Notably, the first patient, who had recently undergone laparoscopic right duplex nephrectomy, presented with microscopic hematuria, whereas the other two did not show any gross or microscopic hematuria. Computed tomography urography revealed hydronephrosis and infection without significant renal pelvic space-occupying lesions, with persistently elevated white blood cell (WBC) counts, but no fever. These atypical clinical presentations confounded clinicians, delaying the diagnosis of upper tract urothelial carcinoma until postoperative pathological examination for the first two patients and resulting in advanced-stage diagnosis for the third patient. Postoperative pathology confirmed high-grade invasive upper tract urothelial carcinoma in all three patients.

**Conclusion:**

Upper tract urothelial carcinoma can manifest atypically without hematuria and may be challenging to visualize on computed tomography urography, potentially leading to misdiagnosis. Therefore, clinicians should maintain a high level of suspicion for malignant tumors when patients exhibit hydronephrosis, infection on imaging, and persistently elevated white blood cell counts without fever, even in the absence of typical signs of upper urothelial carcinoma on computed tomography urography.

## 1 Introduction

Upper tract urothelial carcinoma is relatively rare in clinical practice, accounting for only 5%-10% of urothelial carcinomas (1). It mainly occurs in individuals aged 70-90 years, with a higher incidence in men than in women (2). The most prevalent symptoms include painless gross or microscopic hematuria (70–80%) and flank pain (20-40%) (2). Computed tomography urography has the highest clinical value and highest accuracy in the diagnosis of UTUC (3). It provides crucial information about the tumor location, invasion depth, and its relationship with the surrounding structures. Enhanced scanning further aids in understanding the tumor’s blood supply and confirming its nature as a primary option (4). The main CTU appearances of UTUCs include mild enhancement of soft tissue lesions, renal pelvic filling defects, diffuse thickening of the renal pelvic wall, the stipple sign, or phantom calyx (5). However, UTUC can sometimes masquerade with symptoms and signs resembling hydronephrosis and infection, whereas space-occupying findings are atypical in CTU, easily leading to a delay in proper diagnosis and treatment. This study aimed to analyze the clinical manifestations, diagnosis, and treatment of three patients with UTUC who were initially missed because of atypical symptoms and the limitations of CTU in accurately identifying lesions.

## 2 Case presentation

### 2.1 Case 1

**Figure 1:**
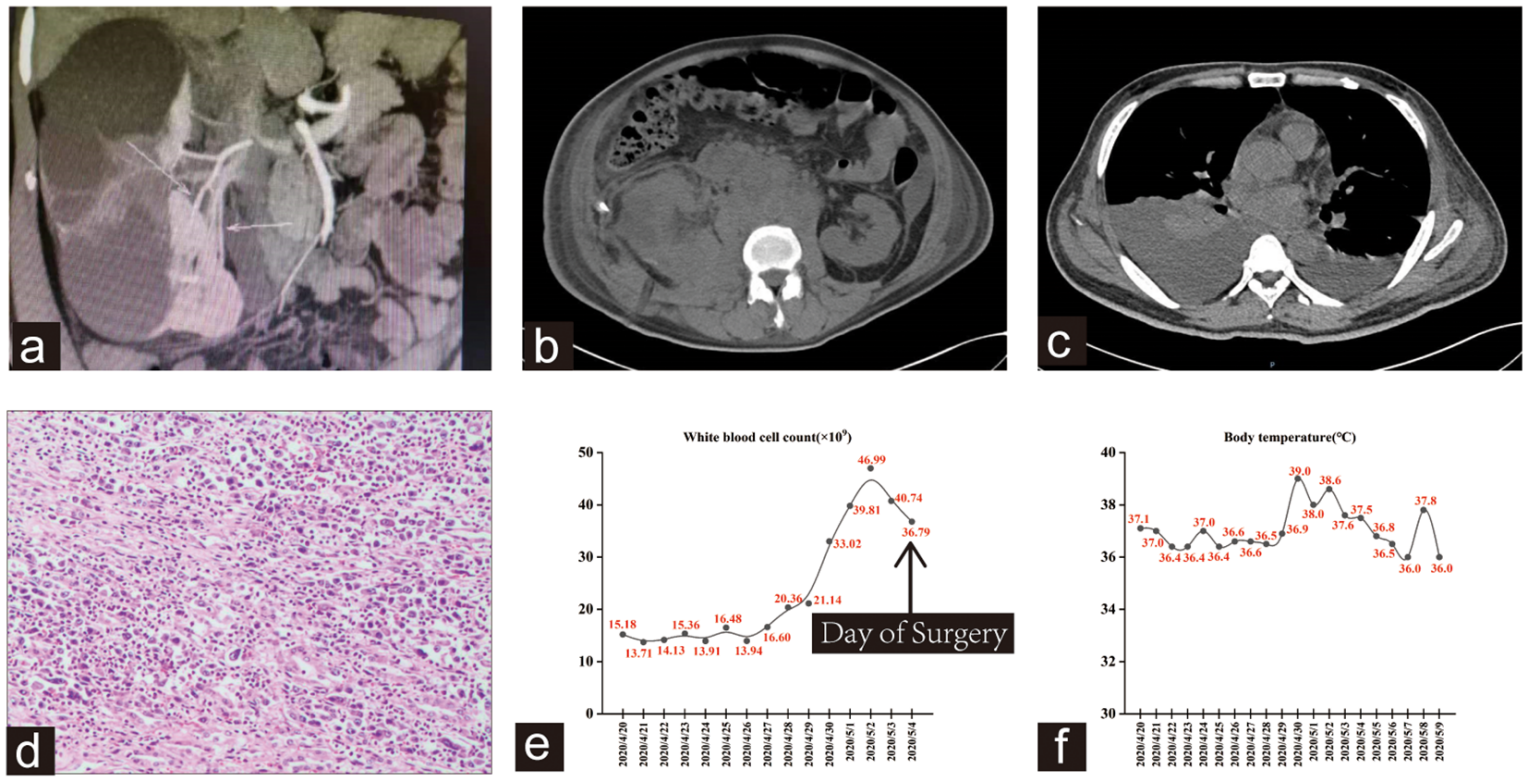
**(A)** CTU shows the right duplex kidney was accompanied by severe upper group hydronephrosis with minimal contrast uptake. **(B)** High density shadow in the operation area was considered as hematoma. Perinephric and retroperitoneal soft tissue shadow, extensive lymphadenopathy. **(C)** Bilateral pleural effusion with atelectasis, particularly on the right side. **(D)** Hematoxylin-eosin staining revealed a high-grade invasive urothelial carcinoma with extensive necrosis. **(E, F)** Trends in this patient’s WBC count and body temperature during hospitalization.

On April 4, 2020, a 49-year-old non-smoker male underwent laparoscopic right duplex nephrectomy at another hospital because of a right duplex kidney with severe hydronephrosis (**Figure 1A**). Following surgery, a right retroperitoneal drainage tube was placed, and on April 20, 2020, the patient presented to our hospital with dark red fluid in the perirenal drainage, accompanied by poor appetite and right flank pain, but without fever. Physical examination revealed anemia, and discharge of approximately 300ml of dark red bloody fluid from the drainage tube. Subsequent computed tomography scans revealed a high-density shadow in the operative area, indicative of hematoma (**Figure 1B**). Upon admission, the patient exhibited a white blood cell count of 15.2×10^9^/L and a neutrophil count of 12.6×10^9^/L. Urinalysis indicated 133.3 red blood cells/µL.

Despite receiving blood transfusion, anti-infective therapy, and hemostatic management, the patient’s condition did not improve. On April 24, 2020, the patient underwent right renal exploration and hematoma evacuation, after which he developed tachypnea and elevated WBC count. A follow-up CT scan revealed bilateral pleural effusion with lung tissue atelectasis, along with extensive perirenal and retroperitoneal lymphadenopathy on the right side, as well as abdominal and pelvic effusion (**Figure 1C**). Subsequent procedures, including right thoracic drainage and abdominal puncture and drainage, did not halt the deterioration of the patient’s condition, and he progressed to septic shock on May 4, 2020. After a specialist multidisciplinary team meeting, a systemic infection resulting from surgical wound exudate was considered. Consequently, the patient underwent right nephrectomy and debridement, but his WBC count remained elevated. Further examination of the bone marrow and peripheral blood revealed altered granulocyte infection, decreased erythrocyte proportion, and hemophagocytosis. Histopathological examination revealed a high-grade invasive urothelial carcinoma with extensive necrosis (**Figure 1D**). The tumor invaded the renal capsule, cortex, and sinus adipose tissue. On the evening of May 09, 2020, the patient experienced ventricular tachycardia and cardiac arrest, ultimately succumbing to his condition after ineffective resuscitation. As indicated in **Figures 1E, F**, the patient initially presented with normal body temperature upon hospital admission. However, as the disease progressed and multiple organ dysfunction syndrome developed in the later stages, significant fluctuations in body temperature and fever were observed.

### 2.2 Case 2

**Figure 2:**
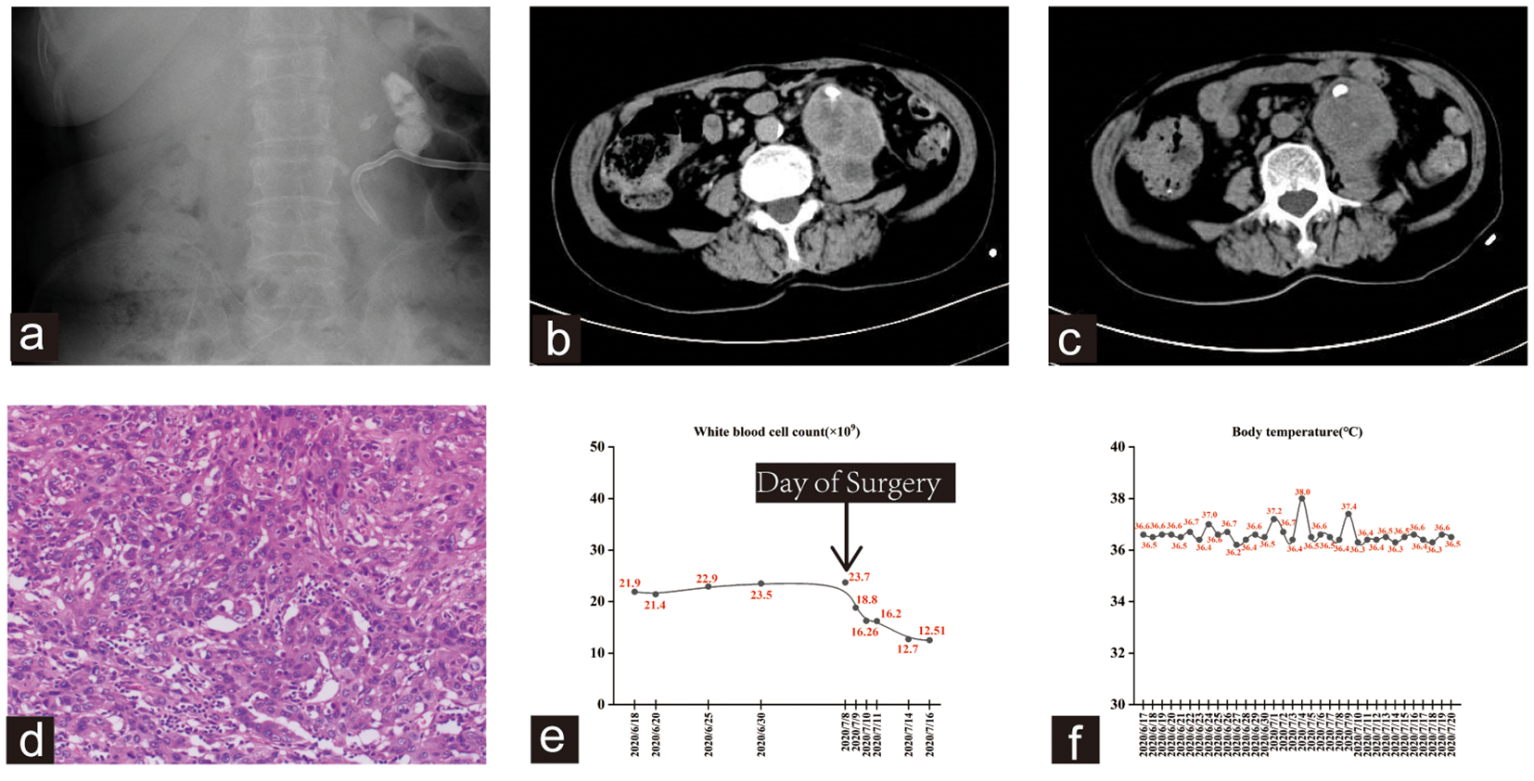
**(A)** kub showed multiple stones in the left kidney and ureter. A left renal drainage tube was seen. **(B, C)** The CTU scan showed an unclear boundary between the left kidney and the adjacent psoas major muscle, with both structures displaying uneven enhancement. Additionally, there was evidence of malrotation in the right kidney. **(D)** The hematoxylin and eosin staining revealed high-grade invasive urothelial carcinoma with extensive necrosis and focal sarcomatoid differentiation. **(E, F)** Trends in WBC count and body temperature of the patient.

A 74-year-old non-smoking female was admitted to our hospital because of intermittent left flank pain for nearly 2 months. She had a medical history of left renal tuberculosis, which was successfully treated with medical intervention 40 years previously. One month prior to her current admission, she was diagnosed with left-sided pyonephrosis at another hospital. Her symptoms improved after undergoing a left renal nephrostomy and anti-infection treatment. However, 10 days after discharge, the pain recurred. Subsequent outpatient examination revealed multiple stones in the left kidney and ureter (**Figure 2A**). Routine blood examination upon admission indicated a WBC count of 21.90×10^9^/L with a neutrophil count of 19.1×10^9^/L and an erythrocyte sedimentation rate of 87 mm/h. CTU revealed an indistinct demarcation between the left renal and adjacent psoas major muscle, with the identification of a left-sided psoas abscess (**Figures 2B, C**).

The patient underwent a percutaneous puncture and drainage of the abscess. However, bacterial culture, acid-fast staining, and fungal examination of blood and drainage yielded negative results. As a result, bone marrow and peripheral blood were evaluated, showing hyperplastic anemia in the bone marrow. Emission computed tomography (ECT) revealed a total glomerular filtration rate (GFR) of 108.0 ml/min, with the left at 6.1 ml/min and the right at 101.8 ml/min. Subsequently, the patient underwent left nephrectomy and psoas abscess resection, and histopathological examination revealed a high-grade invasive urothelial carcinoma with extensive necrosis and local sarcomatoid differentiation (**Figure 2D**). This invasive carcinoma extensively invaded most of the kidney and psoas muscle tissues, with evident vascular tumor emboli and nerve invasion. Despite the diagnosis, the patient declined subsequent adjuvant therapy and unfortunately succumbed to disease progression six months later. Notably, the patient exhibited persistent leukocytosis without fever, which significantly decreased after left nephrectomy. This observation is shown in **Figures 2E, F**.

### 2.3 Case 3

**Figure 3:**
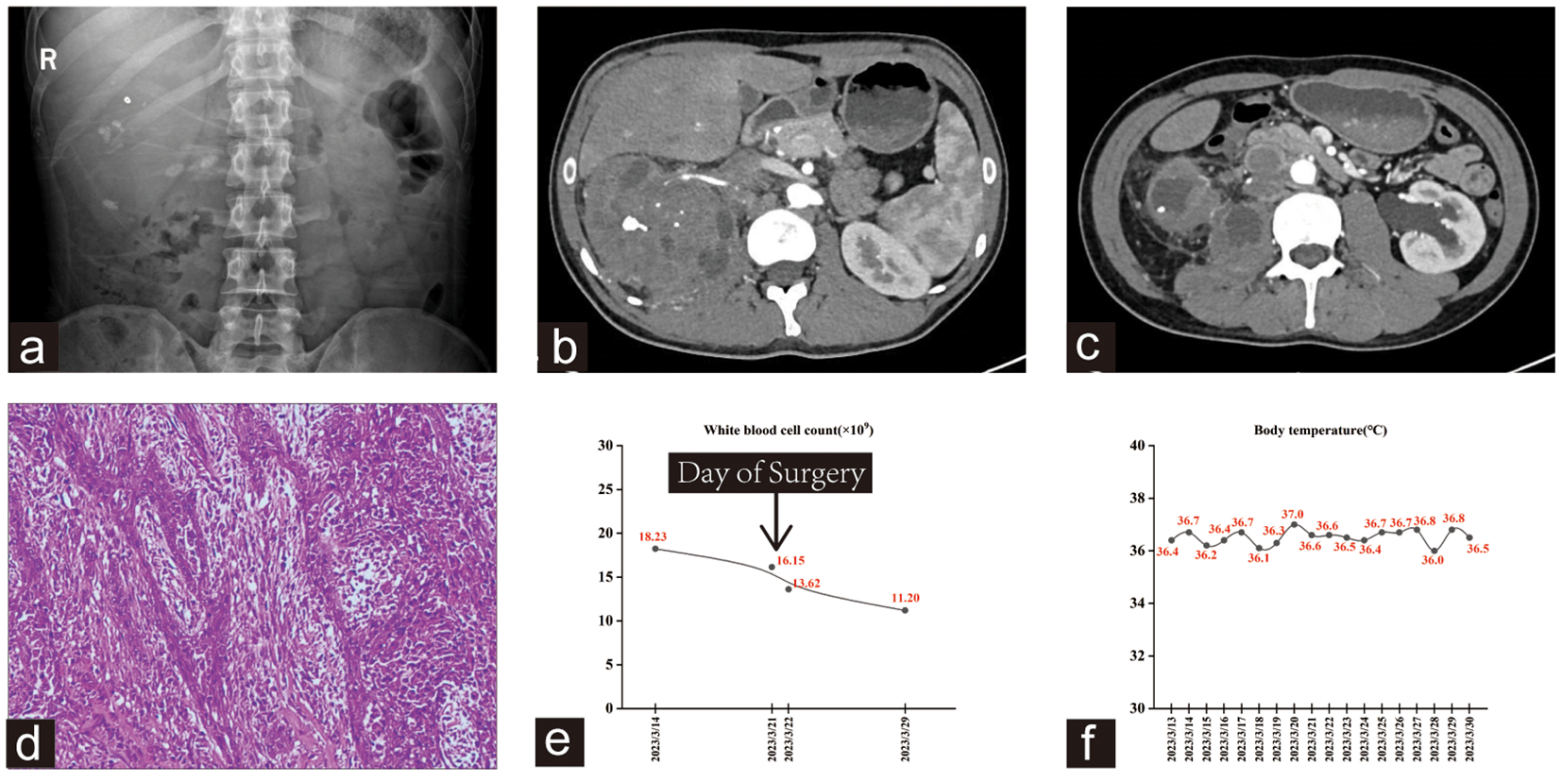
**(A)** kub showed multiple stones in both kidneys. **(B, C)** The right kidney had a mixed density mass involving the right psoas major muscle and retroperitoneal space, with multiple small blood vessels passing through it. **(D)** Hematoxylin-eosin staining showed high-grade invasive urothelial carcinoma with glandular and squamous differentiation. **(E, F)** Trends in this patient’s WBC count and body temperature during hospitalization.

A 33-year-old male with no history of smoking presented to our outpatient clinic with flank pain and anorexia on March 13, 2023. Bilateral renal calculi were found on outpatient examination (**Figure 3A**). The patient underwent a sequential right percutaneous nephrolithotomy and right ureteroscopic holmium laser lithotripsy between September 2017 and April 2022. Upon admission, the WBC count was 19.59×10^9^/L, and the neutrophil count was 13.38×10^9^/L. CTU revealed a mixed-density mass in the right kidney along with bilateral pyelectasis, hydronephrosis, and multiple stones in both kidneys (**Figure 3B**). Enlarged retroperitoneal paracaval lymph nodes were also observed (**Figure 3C**). Furthermore, an ECT examination revealed a total GFR of 51.06 ml/min, with 41.08 ml/min contributed by the left kidney and 9.98 ml/min from the right kidney. Positron emission tomography-computed tomography revealed a right renal mass invading the adjacent psoas major muscle, and abnormally increased metabolic activity in multiple enlarged lymph nodes in the right renal hilar and retroperitoneal areas. Notably, the patient’s medical history revealed slightly enlarged lymph nodes, initially discovered in September 2017, in the retroperitoneal para-vena cava and mesenteric region. Subsequently, the patient revisited our hospital multiple times because of renal or ureteral calculi that caused hydronephrosis. Reexamination of the CTU revealed multiple enlarged lymph nodes, with no evidence of hematuria or renal pelvic lesions. The tumor could not be detected or diagnosed earlier partly owing to interference from hydronephrosis and infection.

Open right RNU was performed on March 21, 2023, and subsequent histopathological examination revealed a high-grade invasive urothelial carcinoma with glandular and squamous differentiation (**Figure 3D**). The tumor had invaded the renal sinus fat and parenchyma, as well as the para-aortic and right renal hilar lymph nodes, with an extensive intravascular tumor thrombus. Subsequently, gene detection using next-generation sequencing (NGS) was performed at an outside referral institute, yielding results that indicated a low tumor mutational burden with no mutation in genes that impair DNA mismatch repair (MMR). Similar to the other two cases, this patient exhibited a markedly elevated WBC count, whereas his temperature remained normal (**Figures 3E, F**). This patient experienced satisfactory postoperative recovery, and subsequent re-examination led to the determination that adjuvant treatment should commence four weeks after the operation. Combined therapy with gemcitabine (1,700 mg on days 1 and 8, q3w) and Toripalimab (240 mg on day 1, q3w) was administered in April 2023, with a total of four treatment cycles. He recovered well from surgery and continues to live with high quality of life, with no evidence for residual or metastatic disease. Currently, the patient continues maintenance therapy with Toripalimab (240 mg on day 1, q3w) as a monotherapy.

## 3 Discussion

Upper tract urothelial carcinoma (UTUC) is a rare type of genitourinary malignancies, with an estimated annual incidence of 1–2 cases per 100,000 people (6). The diagnosis of UTUC is often made following abdominal-pelvic cross-sectional imaging in response to symptoms such as gross hematuria or flank pain, but is also made in otherwise asymptomatic patients with microhematuria, those with a history of bladder cancer, or less often as an incidental finding (3). Computed tomography urography (CTU) is considered the most valuable and accurate imaging technique (2). A meta-analysis involving 13 studies and 1233 patients demonstrated that CTU had a pooled sensitivity of 92% (95%CI 0.85-0.96) and a pooled specificity of 95% (95%CI 0.88-0.98) for UTUC detection (7). Nevertheless, in some cases, the symptomatology and signs of UTUC may be atypical, and CTU may fail to identify the lesion, resulting in misdiagnosis or missed diagnosis. Currently, the gold standard treatment for non-metastatic UTUC is radical nephroureterectomy with bladder cuff excision (8). Radical nephroureterectomy can be performed via open, laparoscopic, and robotic approaches (9). The existing literature suggests that there is no significant difference in the 2-5-year recurrence-free survival, cancer-specific survival, or overall survival between patients undergoing open or laparoscopic RNU (10). However, laparoscopic bladder cuff excision may result in an increased incidence of intravesical recurrence in patients with advanced-stage disease (8, 11). Given that the rate of bladder recurrence after RNU for UTUC is 22–47%, clinicians have used neoadjuvant therapy (NAC) or adjuvant therapy (AC) to improve the survival outcomes of UTUC (12). Multiple studies have shown that neoadjuvant treatments exhibit promising potential for non-metastatic UTUC, as evidenced by the favorable rates of pathological response and clinical outcomes (13–16). However, to date, no randomized controlled trials have supported its use (16, 17). Compared with neoadjuvant therapy, there is much more evidence showing the benefits of adjuvant chemotherapy and immunotherapy in patients with UTUC. A phase 3 multicenter prospective RCT (n = 261) evaluating the benefit of adjuvant chemotherapy after RNU versus RNU alone reported a significant improvement in disease-free survival and metastasis-free survival after a median follow-up of 30.3 months (18).

In this study, three cases of UTUC were presented, each demonstrating atypical clinical presentations leading to delayed diagnosis and treatment. All three patients presented with flank pain, but none reported a history of fever. The first patient had recently undergone duplication nephrectomy of the right kidney, and on admission, urinalysis revealed 133.3 red blood cells/µL. Moreover, all three patients displayed persistently elevated WBC counts, indicative of hydronephrosis and infection. Notably, the other two patients did not exhibit gross or microscopic hematuria. Despite this, the typical imaging findings of UTUC were not identified in the CTU scans of all three patients. Hence, clinically, the suspicion of UTUC was very low. Although CTU scans of the first and third patients showed retroperitoneal enlarged lymph nodes, the tumor was not detected in time, partly due to interference from factors, such as hydronephrosis or infection. Consequently, the infected or non-functional kidneys were surgically removed, and postoperative pathology revealed high-grade invasive urothelial carcinoma.

UTUC is prone to misdiagnosis of inflammatory kidney diseases such as xanthogranulomatous pyelonephritis (19, 20). This was demonstrated in a case reported by Ordones et al., in which a patient presented with symptoms of flank pain, collapse, and fever, and was initially diagnosed with xanthogranulomatous pyelonephritis (20). Jena et al. reported three cases of UTUC who presented with a low-grade fever and persistent leukocytosis (19). None of these patients had any hematuria and they were diagnosed only on biopsy or on nephrectomy. Similarly, the last two cases mentioned in our study had no hematuria. Moreover, all of them presented with persistent leukocytosis, while none had a history of fever (the first patient presented with significant fluctuations in body temperature as the disease progressed and multiple organ dysfunction syndrome developed). The clinical symptoms of some severe renal inflammatory diseases may mask those of UTUC, potentially leading to misdiagnosis or missed diagnosis. Yeh et al. found that among patients undergoing nephrectomy for nonfunctional kidneys due to urolithiasis with clinical symptoms such as chronic inflammatory irritation and infection, a significant number were subsequently diagnosed with malignancy (21). Notably, in both our study and previous reports, patients who were misdiagnosed had chronic inflammatory irritation and infection, which may not only be a contributing factor to cancer, but may also pose challenges in the diagnosis of UTUC. Urologists should be vigilant about the increased likelihood of neoplasms in patients with long-term infections, and should be more aggressive in ruling out the presence of neoplasms. Under these conditions, urine cytology may be helpful in improving diagnosis rates.

Genomic characterization of UTUC plays a crucial role in identifying tumors associated with Lynch syndrome (LS) and informing personalized treatment strategies. Lynch syndrome (also known as HNPCC, hereditary non-polyposis colorectal cancer) is an autosomal dominant genetic syndrome that results in a wide spectrum of malignancies due to genetic mutations that impair DNA mismatch repair (MMR) (22). UTUC is the third most common malignancy in Lynch syndrome (23). Up to 21% of UTUC cases may be linked to unrecognized LS (23). Furthermore, multiple clinical trials have shown that tumors with deficient MMR exhibit significantly higher response rates to immune checkpoint inhibitors than those with intact MMR expression, providing compelling evidence for personalized treatment approaches in such cases (24, 25). The final patient in this report underwent gene detection through next-generation sequencing, which revealed no mutations in genes that impair DNA mismatch repair. Tumor-associated leukocytosis, a paraneoplastic syndrome occasionally observed in patients with urothelial carcinoma, has a reported prevalence of approximately 0.6% (26). This condition is associated with the secretion of granulocyte colony-stimulating factor by neoplastic cells, indicating advanced urothelial carcinoma and unfavorable prognosis (26, 27). Notably, the patients in our study presented with elevated WBC counts and normal body temperature, which were initially thought to be caused by infection. Subsequently, we performed antibiotic treatment and bone marrow biopsy. However, their remarkable degree of leukocytosis could not be explained by infection or hematological diseases, suggesting the possibility of UTUC with paraneoplastic syndrome.

## 4 Conclusion

In conclusion, UTUC sometimes lacks typical manifestations and only presents with hydronephrosis on imaging and persistent leukocytosis without fever, which can easily lead to missed diagnosis. Therefore, clinicians should be highly alert to the possibility of urothelial cancer with paraneoplastic leukocytosis, pay attention to differential diagnosis, and avoid missed diagnosis and misdiagnosis, even if CTU does not show typical signs of UTUC. Our study contributes to improving the diagnostic ability of UTUC and promotes understanding of this disease.

## Data availability statement

The data supporting the findings of this study are available from the corresponding author upon reasonable request.

## Ethics statement

The study was approved by the Ethics Committee of the Zhongnan Hospital of Wuhan University. Written informed consent was obtained from each individual(s) for publication of potentially identifiable images or data included in this article.

## Author contributions

LG: Data curation, Formal analysis, Validation, Visualization, Writing – original draft, Writing – review & editing. XB: Formal analysis, Writing – review & editing. KT: Formal analysis, Writing – review & editing. XW: Validation, Visualization, Writing – review & editing. TL: Conceptualization, Formal analysis, Project administration, Validation, Visualization, Writing – review & editing.
